# Aeroacoustics and psychoacoustics characterization of a boundary layer ingesting ducted fan

**DOI:** 10.1038/s44384-025-00010-z

**Published:** 2025-05-15

**Authors:** Feroz Ahmed, Carlos Ramos-Romero, Antonio J. Torija, Mahdi Azarpeyvand

**Affiliations:** 1 Department of Aerospace Engineering, University of Bristol, Queen’s Building, Bristol, BS8 1TR UK; 2 Acoustics Research Centre, University of Salford, 43 Crescent, Salford, M5 4WT Greater Manchester UK

**Keywords:** Energy science and technology, Psychology, Psychology, Applied physics, Acoustics, Engineering, Aerospace engineering

## Abstract

A comprehensive wind tunnel investigation was conducted to analyze noise generation, propagation, and perception mechanisms in a boundary layer ingesting (BLI) ducted fan through integrated aeroacoustic and psychoacoustic assessments. The study examines interactions between an incoming adverse pressure gradient turbulent boundary layer flow, developed over a curved wall, and the ducted fan. The fundamental investigation confirms that the fan thrust regime influences aerodynamic, aeroacoustic, and psychoacoustic characteristics, exhibiting various haystacking phenomena. High-thrust operation induces a pronounced upstream suction effect, accelerating the boundary layer flow, amplifying bulk momentum, and intensifying turbulence ingestion, leading to fan aeroacoustics and associated fan haystacking in noise spectrum. In contrast, low-thrust operation minimally alters the boundary layer flow, with reduced suction and noise dominated by duct aeroacoustics and the associated duct haystacking due to interactions between ingested turbulence and the duct’s acoustic field. The psychoacoustic assessments indicate that both fan and duct haystacking contribute to higher perceived noise in the high- and low-thrust regime, respectively.

## Introduction

According to the European Commission’s Flight Path 2050 (FP2050) challenge, by 2050, future commercial transport aircraft should reduce CO_2_ emissions by 75%, NO_*x*_ emissions by 90%, and noise emissions by 65%^1^. These targets reflect a shift towards more sustainable future flight technologies, with an emphasis on minimizing the environmental footprint of aviation. To meet these ambitious targets, the airframe architecture and propulsion systems must undergo radical changes, driving the need for green aviation technologies. One promising approach to tackle these challenges is the transition towards electric propulsion systems, including the novel electric aircraft concepts within the urban air mobility (UAM) sector, particularly electric vertical take-off and landing (eVTOL) vehicles. The electric propulsion system concepts being developed for future aircraft include distributed electric propulsion (DEP) systems and ducted fan systems, both of which have the potential to meet these stringent environmental goals.

While electric-powered UAM systems offers reduced carbon emissions compared to fossil fuel-powered conventional aircraft, the challenge of noise emission remains unresolved. The psychoacoustic annoyance associated with UAM systems, due to their frequent operations in urban areas, impacts human health and disrupts wildlife. Therefore, despite advancements in emission reductions, the aviation industry must prioritize addressing the urgent issue of noise, which poses a major obstacle in obtaining vehicle certifications. Developing technologies to reduce noise levels and annoyance is essential to ensure the broader acceptance and success of new aircraft technologies, including UAM systems.

**Fig. 1:**
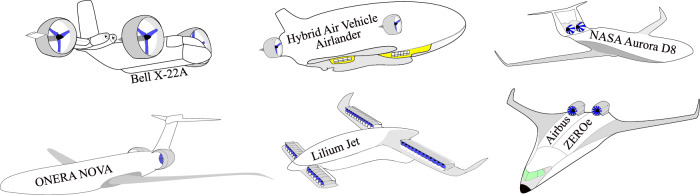
Schematic of novel aircraft featuring boundary layer ingesting (BLI) ducted fan propulsion systems.

Fan system noise is one of the major sources of noise in the future aircraft concepts, including the vehicles with DEP and ducted fan systems. This noise is a widespread industrial concern, not only in the aerospace^2–4^ but also in the maritime^5^ sectors. A major contributor to this noise is the turbulence ingestion noise (TIN), which poses several design challenges for rotating fans. The search for propulsion systems with zero emission has shown renewed interest in electric ducted fans, as demonstrated by projects such as, Embraer X, Airbus ZEROe, Green Jet, and Hybrid Air Vehicle (see Fig. 1). These ducted fan propulsion systems are favored for their potential to achieve zero emissions compared to conventional non-ducted (or open) fan systems.

The ducted fans are generally mounted in two primary configurations, i.e., isolated (or podded) when mounted away from the airframe body and installed (or buried or embedded) when mounted close to or partially buried within the airframe body^6^. Recent developments in the installed ducted propulsion system have particularly focused on the implementation of boundary layer ingesting (BLI) ducted fans in next-generation electric/hybrid aircraft. These installed BLI ducted fan systems are partially embedded into the airframe, strategically designed to ingest incoming turbulent boundary layer flows, thereby optimizing aerodynamic efficiency^7^. The examples of large-scale transport aircraft featuring installed BLI ducted fan propulsion systems include the ONERA NOVA^6,8,9^, NASA/MIT Aurora D8, Airbus Nautilus, and MIT SAX-40 (see Fig. 1). In the Urban Air Mobility (UAM) sector, the Lilium Jet^10^ is an example of small-scale electric vertical take-off and landing (eVTOL) aircraft featuring installed BLI ducted fan propulsion systems (see Fig. 1). Although BLI ducted fan systems contribute to reduced fuel consumption, a critical benefit for sustainable aviation, they cause an alteration in the noise signature, influenced by the upstream airframe design. Consequently, these configurations require a comprehensive analysis of noise generation mechanisms and their impact on the auditory comfort of passengers and nearby communities.

**Fig. 2:**
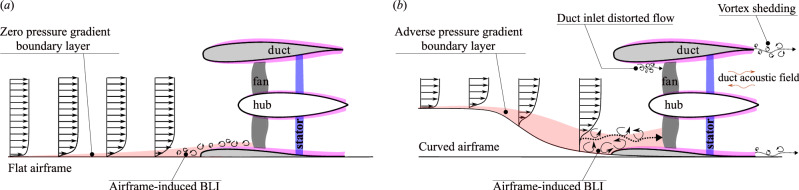
Ducted fan ingesting boundary layer flows. **a** Zero pressure gradient and (**b**) adverse pressure gradient flow^11^. The boundary layer develops over a curved airframe that resembles the rear fuselage of the ONERA NOVA and NASA Aurora D8 aircraft concepts featuring boundary layer ingesting (BLI) ducted fans (see Fig. 1).

The noise signatures in BLI ducted fan propulsion systems are quite complex, which arise due to several factors^3,11–16^ (see Fig. 2): (i) the rotating fan generating multiple frequencies (i.e., blade-passing frequency tones and its harmonics), (ii) the duct acoustic field^15,17,18^ interacting with periodic flow disturbances (i.e., flow harmonics), (iii) position of fan within ducts, (iv) the blade tip vortices interacting with duct boundary layer, (v) fan–stator interaction^19^, and (vi) the incoming turbulent flow interacting with the rotating fan and the duct acoustic field. Significant attention has been directed towards the investigation of turbulence-ingestion noise (TIN) generated from fans/propellers/rotors, encompassing both open (or non-ducted) and ducted (or shrouded) configurations. This comprehensive body of research has explored the aeroacoustics of fans ingesting various types of incoming turbulent flows, including: (i) atmospheric turbulence, with investigations in both open^2,20–24^ and ducted^2,25^ configurations, (ii) turbulent wakes, studied in both open^26–28^ and ducted^2^ configurations, and (iii) turbulent boundary layer ingestion (BLI), examined in both open^5,28,29^ and ducted^8,9,16,30–33^ configurations.

Most BLI research focuses on the noise characteristics of open propellers/rotors/fans ingesting zero pressure gradient turbulent boundary layers developing over flat surfaces^2,5^ (see Fig. 2a). Limited noise study exists on BLI ducted fans, particularly in the context of adverse pressure gradient turbulent boundary layers developing over curved airframe surfaces^4,11^ (see Fig. 2b). Addressing this gap, Ahmed et al.^11^ investigated the fundamental mechanisms of noise generation and propagation in BLI ducted fans subjected to an adverse pressure gradient boundary layer developed over a curved S-plate wall, resembling the rear fuselage of the ONERA NOVA and NASA Aurora D8 aircraft concepts (see Fig. 1).

The complex noise signatures generated by BLI ducted fan systems require a comprehensive analysis considering different relevant psychoacoustic features with a high influence on noise annoyance. Focusing solely on the noise spectral features and overall sound pressure levels might not provide the whole picture of how BLI ducted fan noise would be perceived. For example, tonal components can exhibit spectral broadening and hump features due to haystacking phenomena across different thrust regimes, as described by Ahmed et al.^11^. Such spectral features can significantly affect the perceived annoyance levels experienced by communities exposed to these noise sources. Despite the increasing interest in BLI ducted fans for next-generation aircraft, the psychoacoustic implications of their noise emissions remain largely unexplored, highlighting a critical research gap. Addressing this knowledge gap requires an integrated study that establishes the relationship between aerodynamics, aeroacoustics, and psychoacoustics in these systems. This paper focuses on bridging this knowledge gap.

The purpose of this study is to comprehensively study the relationship among noise generation, far-field noise radiation, and annoyance in a BLI ducted fan by extending the previous study of Ahmed et al.^11^. By gaining an in-depth understanding of the mechanisms of noise generation, radiation, and perception in the BLI ducted fans, it is hoped that the industrial guidelines can be developed for quieter and less annoying airframe-integrated propulsion systems in the future aircraft concepts.

The structure of the paper is as follows. The section “Methods" details the wind tunnel experimental setup, the methodology employed, and the diagnostic tools utilized for analysis. The section “Results and discussions" provides an in-depth examination of findings, covering aerodynamic, aeroacoustic, and psychoacoustic assessments comprehensively.

## Results and discussion

This section presents the experimental setup, measurement equipment, data acquisition techniques, and analysis framework, followed by an in-depth discussion of the results. A comprehensive investigation of the BLI ducted fan is conducted, focusing on three key aspects: aerodynamics, aeroacoustics, and psychoacoustics. The findings offer critical insights into noise generation, propagation mechanisms, and perceptual characteristics, providing a deeper understanding of BLI ducted fan systems for practical applications.

### Experimental set-up

**Fig. 3:**
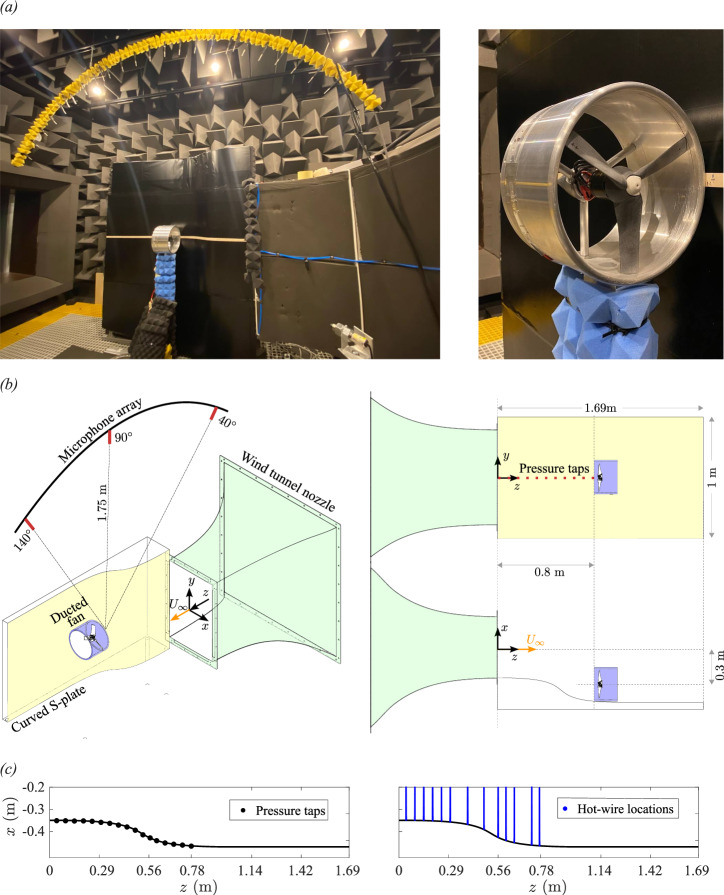
Measurement set-up overview. **a** Boundary layer ingesting (BLI) ducted fan test rig inside the anechoic chamber of the aeroacoustics wind tunnel facility at the University of Bristol. **b** Schematic representation of the experimental setup, illustrating the positioning of the curved S-plate, ducted fan, and microphone array; and (**c)** Measurement layout on the curved S-plate, showing the distribution of pressure taps along the streamwise direction and a dense grid of hot-wire measurement points (~150 points per streamwise location) for detailed velocity field analysis.

An extensive experimental test campaign was conducted in the University of Bristol’s aeroacoustics wind tunnel facility to understand the flow characteristics, far-field noise radiation, and annoyance characteristics of the BLI ducted fan. Figure 3 illustrates the measurement setup used for these experiments. The following text describes the experimental setup, equipment, and measurement techniques employed in the study, which follows a similar test rig design to that considered by Ahmed et al.^11,34–36^. A wide range of data are captured, including fan loading, velocity and pressure field distribution along the curved S-plate, and radiated noise in the far field.

The test rig comprises an electric ducted fan mounted next to a curved wall (see Fig. 3b). The BLI ducted fan test is designed and developed to demonstrate the partially-buried engines in the aerospace sector, e.g., the propulsion unit in the ONERA NOVA configuration (see Fig. 1). The ducted fan, based on the Bell X-22A design^37^ (see Fig. 1), utilizes a 3-bladed fan mounted inside a straight duct with a constant cross-sectional area. The duct is manufactured from CNC machined aluminum, while the fan is manufactured using rapid prototyping with multi-jet fusion technology and nylon PA-12 material to maintain strength during the experiments. The fan, designed with NACA-23012 profile, has a diameter (*D*) of 254 mm and a pitch-to-diameter ratio of 0.85. The geometrical parameters of the NACA23012 blade profile were taken from Mort and Gamse^37^, as highlighted in Ahmed et al.^11^.

Additionally, a curved S-plate wall set-up with streamlined wall curvature is designed to generate an adverse pressure gradient turbulent boundary layer flow (see Fig. 3b). This S-plate setup represents a portion of the fuselage where the ducted fan is typically embedded in the ONERA NOVA configuration (see Fig. 1). The S-plate profile features a convex wall in the upstream region, a concave wall in the intermediate region, and a flat wall in the downstream region. The curvature of the S-plate is mathematically defined using a cubic Bezier function, thereby introducing a geometrical gradient across its length. When the flow is traversed along the S-plate, an adverse pressure gradient turbulent boundary layer flow is generated in the concave region. This is discussed in detail by Ahmed et al.^11^.

The measurements on the ducted fan configurations were carried out in the aeroacoustics wind tunnel facility of the University of Bristol (see Fig. 3a, b), which features a closed-circuit open-jet anechoic wind tunnel with an open test section of length 2 m and a nozzle exit of 0.5 m × 0.775 m with a contraction ratio of 8.4:1. With turbulence levels as low as 0.1%, the wind tunnel is capable of achieving free-stream velocities up to 40 m/s. All exposed surfaces within the anechoic chamber, including the contraction nozzle, are lined with acoustic foam wedges to fully absorb sound reflections. According to the ISO 3745 standardized testing procedure for both pure tone and broadband testing, the anechoic chamber allows anechoic measurements down to 160 Hz. Mayer et al.^38^ provide more details on this aeroacoustic facility.

The hub of the ducted fan houses an electric motor and load cell. The fan was powered by an AT4125 T-MOTOR, and aerodynamic loads were measured using a six-axis ATI F/T sensor (Mini 40) load cell. The calibration of the load cell was verified with known weights prior to the tests, and zero-bias adjustments on the load cell were made under no axial inflow and no fan rotational speed. The curved S-plate wall was instrumented with 23 static pressure taps along its midspan, maintaining a consistent tap-to-tap distance of 5 mm (see Fig. 3c and Ahmed et al.^11^). These taps were distributed as follows: 6 in the convex upstream region, 8 in the concave intermediate region, and 8 in the flat downstream region. Additionally, a probe was installed beneath the nozzle to record the ambient static pressure. To measure the static pressure, a MicroDaq pressure scanner from Chell Instruments was used. This pressure scanner had a capacity of 1psi and provided measurements with an accuracy of 0.05%. It is important to note that the pressure tap readings were zeroed before the initiation of flow.

Hot-wire measurements were conducted to assess the velocity field in the crosswise direction at various streamwise positions along the curved S-plate (see Fig. 3c). The data were collected using a Dantec 55P16 single-wire probe featuring a platinum-plated tungsten wire with a diameter of 5 μm and a length of 1.25 mm, connected to a Dantec StreamLine Pro CTA system. For precise and automated positioning, the probe was mounted on a Thorlabs LTS300M 2D traverse system with a positioning accuracy of ±5 μm. At each streamwise location on the curved S-plate, hot-wire measurements were conducted across a dense grid, comprising approximately 150 points in the crosswise direction to capture detailed flow structures. The probe was carefully calibrated each day of the test campaign using a Dantec 54H10 calibrator to ensure measurement reliability, with uncertainties within ±1%. Measurements were recorded at each location along the S-plate, starting from the closest point of *x* = 1.5 mm from the wall, with high-resolution points concentrated in the crosswise direction near the surface.

The far-field noise measurements on the ducted fan configurations were captured using quarter-inch GRAS 40PL piezoelectric free-field microphones, which have a high dynamic range of 32–150 dB(A) and a flat frequency response from 10 Hz to 10 kHz^38^. The microphones were arranged in a circular array, with each microphone positioned at an angular location (*θ*) relative to the fan axis, measured from the duct inlet plane at a distance of 1.75 m. The microphones were spaced at 5° intervals, covering an angle range from 40° to 140° (see Fig. 3b). The microphone angles of *θ* < 90° correspond to upstream positions, while *θ* > 90° correspond to downstream positions. To ensure the accuracy of the testing, each microphone was calibrated prior to the test using a GRAS 42AA piston phone calibrator.

The measurement data were collected using PXIe-4499 modules housed in a PXIe-1062Q chassis by National Instruments. For the load, hot-wire, and far-field noise measurements, the data were captured at a sampling frequency of 2^16^ Hz for a duration of 32 s, providing high-resolution temporal data for analysis. To achieve accurate pressure distribution results, the surface static pressure measurements were captured at a sampling frequency of 400 Hz for a duration of 32 s.

### Analysis framework

The placement of a ducted fan on the curved S-plate wall (see Fig. 3b) was determined based on the flow characteristics observed from the S plate. During wind tunnel tests, a high adverse pressure gradient boundary layer flow was observed in the concave region of the S-plate, specifically at a position *z* = 0.8 m downstream of the nozzle exit. The ducted fan was mounted at this specific region of high adverse pressure gradient, signifying ingesting highly three-dimensional distorted flow. This is discussed in detail by Ahmed et al.^11^. The following text discusses the test matrix and methodology adopted in this study.

The fan rotational speed (*N*) is associated with the blade tip Mach number, defined as

1 $${M}_{{\rm{tip}}}=\frac{\pi nD}{{a}_{0}},$$

where *n*(=*N*/60) represents the fan rotational speed in revolutions per second, and *a*_0_ (=343 m/s) the speed of sound in air.

The aerodynamic thrust generated by the fan varies across different flight regimes during its operation. The thrust (*T*) data, obtained from the load cell, is expressed in terms of non-dimensionalized coefficient of thrust, defined as

2 $${C}_{{\rm {T}}}=\frac{T}{{\rho }_{\infty }{n}^{2}{D}^{4}},$$

where *ρ*_*∞*_ (=1.225 kg/m) is the flow density.

The thrust regime during flight is characterized by the advance ratio (*J*), which relates the flight speed (or axial inflow velocity) (*U*_*∞*_) to the blade tip speed

3 $$J=\frac{{U}_{\infty }}{nD}.$$

**Fig. 4:**
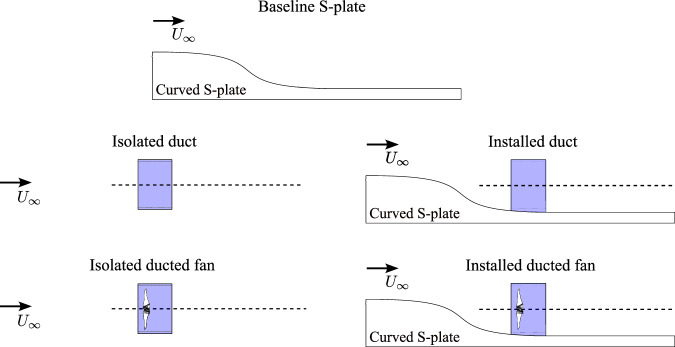
Experimental source decomposition approach adopted in this study.

The aeroacoustics of a BLI ducted fan system are influenced by the fan, duct, and curved S-plate. Two configurations of ducted fan systems were considered in this study, namely, the isolated (without S-plate) and the installed (with S-plate) ducted fans (see Fig. 4 and Table 1). To characterize duct acoustics, two additional configurations were further considered, namely, the isolated (without S-plate) and the installed (with S-plate) duct (see Fig. 4 and Table 1). It is important to note that both the fan and strut components were removed from the isolated and installed duct configurations.

**Table 1:** Summary of test configurations, as illustrated in Fig. 4

Configuration	Description	*N* (rpm)	*M*_tip_	*U*_*∞*_ (m/s)	Thrust level
Baseline S-plate	S-plate	–	–	8-30	–
Isolated duct	Duct	–	–	8–30	–
Installed duct	Duct & S-plate	–	–	8–30	–
Isolated ducted fan	Ducted fan	6000	0.23	8–30	High–Low
		11,000	0.43	8–30	High–Low
Installed (BLI) ducted fan	Ducted fan & S-plate	6000	0.23	8–30	High–Low
		11,000	0.43	8–30	High–Low

Key parameters include fan rotational speed (*N*), blade tip Mach number (*M*_tip_), and flight speed (*U*_*∞*_).

The test campaign was conducted at two fixed fan rotational speeds (*N*) of 6000 and 11,000 rpm, corresponding to blade tip Mach numbers (*M*_tip_) of 0.23 and 0.43, respectively (see Table 1). The aerodynamic thrust generated by the fan was characterized across various flight regimes using the advance ratio (*J*), which relates the axial inflow velocity (*U*_*∞*_) to the blade tip speed (*nD*). For *N* = 6000 rpm, *J* ranged from 0.315 to 1.181, while for *N* = 11,000 rpm, it ranged from 0.172 to 0.6442. These variations in *J* were achieved by adjusting *U*_*∞*_ between 8 and 30 m/s. The lower values of *J* correspond to higher thrust levels, whereas higher values of *J* correspond to lower thrust levels. For *N* = 6000 rpm, the thrust ranges from high thrust at *U*_*∞*_ = 8 m/s and *J* = 0.315 to low thrust at *U*_*∞*_ = 30 m/s and *J* = 1.181. Similarly, for *N* = 11,000 rpm, the thrust varies from high thrust at *U*_*∞*_ = 8 m/s and *J* = 0.172 to low thrust at *U*_*∞*_ = 30 m/s and *J* = 0.6442. This wide range of thrust values covers all flight regimes, ranging from takeoff to cruise.

### Diagnostic tools

To accurately diagnose flow characteristics, noise, and annoyance issues associated with the BLI ducted fan, a wide range of multidisciplinary metrics are employed. The details of these metrics are discussed in the following text.

#### Aerodynamic metrics

The first step in the aerodynamic assessment of the BLI ducted fan is to analyze the boundary layer flow characteristics as it interact with the fan. The flow field distribution along the S-plate is assessed by examining the velocity data captured using hot-wire anemometry and pressure data obtained from static pressure taps (see Fig. 3c).

The hot-wire anemometry data enables the evaluation of the boundary layer development and its interaction with the rotating fan. Velocity measurements are expressed in terms of mean velocity (*u*_mean_) and root-mean-square (rms) velocity (*u*_rms_). The *u*_mean_ characterizes the bulk flow momentum, while *u*_rms_ provide insights into the turbulence levels introduced during the ingestion process. Together, these velocity metrics offer a comprehensive understanding of the boundary layer ingestion process as it interacts with the fan.

The static pressure tap data provides information on the steady pressure distribution along the curved S-plate, which is essential for understanding the pressure gradients influencing boundary layer ingestion. The static pressure tap measurements are expressed in terms of the mean pressure coefficient,

4 $${C}_{{p}_{{\rm{mean}}}}=\frac{{p}_{{\rm{mean}}}-{p}_{\infty }}{0.5{\rho }_{\infty }{U}_{\infty }^{2}},$$

and root-mean-square pressure coefficient,

5 $${C}_{{p}_{{\rm{rms}}}}=\frac{{p}_{{\rm{rms}}}-{p}_{\infty }}{0.5{\rho }_{\infty }{U}_{\infty }^{2}},$$

where *p*_*∞*_ the free stream static pressure, *p*_mean_ and *p*_rms_ the mean and root-mean-squared static pressure, respectively.

#### Aeroacoustic metrics

The aeroacoustic assessment of the BLI ducted fan requires an analysis of the acoustic pressure data obtained from the far-field microphones at different microphone angles (*θ*_*j*_) (see Fig. 3b), which can be expressed in terms of the sound pressure level (SPL), defined as

6 $${\rm{SPL}}({\theta }_{j};f)=10\cdot {\log }_{10}\left(\frac{{\phi }_{{\rm {PP}}}({\theta }_{j};f)\cdot \Delta f}{{p}_{{\rm{ref}}}^{2}}\right),$$

where *ϕ*_PP_ is the ensemble-averaged power spectrum (or power spectral density, PSD) based on Welch’s method, and *p*_ref_ the reference sound pressure for air (20 μPa), and the Δ*f* the frequency resolution.

#### Psychoacoustic metrics

The psychoacoustic assessment of the noise associated with the BLI ducted fan requires incorporating the effects of the human auditory system. Since humans perceive sound differently from microphones, an *A*-weighting filter is applied to account for this difference. This filter accounts for the human ear’s sensitivity by representing the inverted equal-loudness-level contours at 40-phon, as defined by the industry standard ISO 226:2023. This adjustment is expressed in terms of *A*-weighted sound pressure level (SPL_*A*_) as

7 $${{\rm{SPL}}}_{A}({\theta }_{j};f)={\rm{SPL}}({\theta }_{j};f)+{A}_{d}(f),$$

where *A*_*d*_ is the *A*-weighted correction factor.

To quantify the total energy content within the acoustic pressure data, the sound pressure levels are typically integrated over a specified frequency bandwidth of interest using the overall *A*-weighted sound pressure level (O*A*SPL), defined as

8 $${\rm {O}}A{\rm {SPL}}({\theta }_{j})=10\cdot {\log }_{10}\left(\mathop{\sum }\limits_{{f}_{1}}^{{f}_{2}}1{0}^{{{\rm{SPL}}}_{A}({\theta }_{j};f)/10}\right),$$

where *f*_1_ and *f*_2_ are the lower and upper frequencies within the bandwidth of interest. The frequency range from 20 Hz to 20 kHz is commonly used for human perception of sound.

In addition, the energy contained within the acoustic pressure data collected from each microphone was analyzed using a series of sound quality metrics (SQMs) to capture distinct noise perception characteristics of the BLI ducted fan. These psychoacoustic metrics have been widely applied in perception-driven design across various fields, including the automotive industry^39^, wind farms^40^, and more recently in the assessment of advanced air mobility (AAM) noise^41,42^. Table 2 summarizes the units, psychoacoustic effects, and the relevant calculation standards or models for each SQM. To better understand the upper range of each SQM, all SQM time series were processed to obtain their 5th percentiles (5% exceedance levels), a statistical value commonly used in psychoacoustic analysis^43,44^. These percentiles represent the higher range of each SQM time history. The SQMs calculations were performed using HEAD Acoustics ArtemiS SUITE 15.2 software.

**Table 2:** Sound Quality Metrics (SQMs) description.

SQM (units)	Description of the psychoacoustic effect	Stantar/Model
Loudness, *N* (sone)	The sensation value of the human perception of sound volume^43^	DIN45631/A1
Roughness, *R* (asper)	The low-frequency variation of the signal amplitude or frequency^43^	Hearing model
Fluctuation strength, *F* (vacil)	The sensation of very-low modulation frequencies of loudness^43^	Hearing model
Tonality, *T* (tonality units)	The sound is perceived with distinct individual tones or narrow-band noise components^53^	Aures/Terhardt
Sharpness, *S* (acum)	Timbre sensation that measures the dispersion of frequency components of sound, and the presence of high-frequency components relative to low-frequency components^52,53^	DIN45692

To further evaluate the contribution of different psychoacoustic features to noise perception, psychoacoustic annoyance (PA) was also calculated. PA is typically expressed as

9 $$PA=N\left(1+\sqrt{{\gamma }_{0}+\mathop{\sum }\limits_{i=1}^{n}{\gamma }_{i}{w}_{{X}_{i}}^{2}}\right),$$

where *γ* are the nominal weighting coefficients, and ${w}_{{X}_{i}}$ represents the function of each of the *n* SQMs, denoted by *X*_*i*_, included in the model^45^.

A more specific psychoacoustic annoyance model, PA_mod_, optimized for aircraft noise, is provided by More’s formulation. This model was derived from a series of psychoacoustic tests involving 7 experiments, 247 participants, and 123 aircraft noise stimuli^46^. Nonlinear least-squares analysis was used to fit the model and estimate the parameters *γ*_*i*_, where $i\in {\mathbb{Z}}\left[0,5\right]$. More’s model is given as

10 $${P{A}}_{{mod}}=N\left(1+\sqrt{{\gamma }_{0}+{\gamma }_{1}{w}_{{{S}}}^{2}+{\gamma }_{2}{w}_{{{FR}}}^{2}+{\gamma }_{3}{w}_{{{T}}}^{2}}\right).$$

More’s model includes the effects on auditory perception of Loudness (*N*), Roughness (*R*) and Fluctuation strength (*F*), but also places special emphasis on the effects of Sharpness (*S*) and Tonality (*T*), which are intrinsic spectral characteristics for rotating machinery such as open propellers and ducted fans^44,47^. The nominal weighting coefficients for Eq. (10) are: *γ*_0_ = −0.16, *γ*_1_ = 11.48, *γ*_2_ = 0.84, *γ*_3_ = 1.25, *γ*_4_ = 0.29, and *γ*_5_ = 5.49. *N* represents the loudness exceeded 5% of the time, and the *w* functions for each of the SQMs are defined as

11 $${w}_{{S}}=\left\{\begin{array}{ll}0.25\left(S-1.75\right){\log }_{10}\left(N+10\right)\quad &\,{\text{if}}\,S \,>\, 1.75\\ 0\quad &\,{\text{if}}\,S \,<\, 1.75\\ \quad \end{array}\right.$$

12 $${w}_{{FR}}=\frac{2.18}{{(N)}^{0.4}}\left(0.4F+0.6R\right),$$

13 $${w}_{T}^{2}=\left[{\left(1-{{\rm {e}}}^{-{\gamma }_{4}N}\right)}^{2}{\left(1-{{\rm {e}}}^{-{\gamma }_{5}T}\right)}^{2}\right].$$

The following subsections provide an in-depth analysis of the boundary layer ingesting (BLI) ducted fan, focusing on three critical aspects, i.e., aerodynamics, aeroacoustics, and psychoacoustics. The study compares the results of the BLI ducted fan in four dissected configurations (see Fig. 4 and Table 1). These configurations are assessed across a range of advance ratios (*J*) while maintaining constant fan rotational speeds (*N*) of 6000 and 11,000 rpm. The following subsections present detailed analyses establishing relationships among these three aspects of the BLI ducted fan.

### Aerodynamic assessment

#### Velocity field

The following text examines the velocity field structures for high-thrust (*J* = 0.172, *U*_*∞*_ = 8 m/s, *N* = 11,000 rpm) and low-thrust (*J* = 0.644, *U*_*∞*_ = 30 m/s, *N* = 11,000 rpm) BLI cases, compared against the baseline S-plate configuration (without the ducted fan) under similar axial inflow conditions (*U*_*∞*_ = 8 and 30 m/s).

**Fig. 5:**
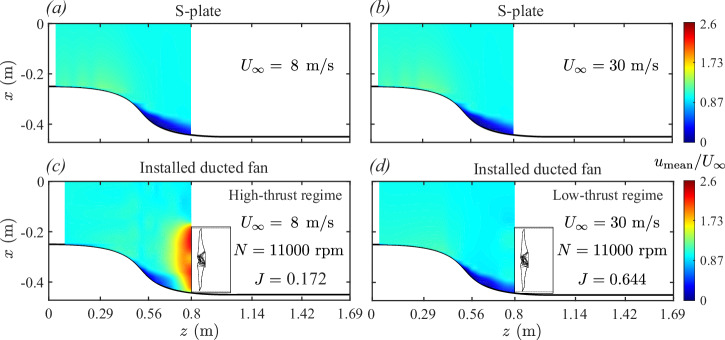
Evolution of the two-dimensional mean velocity field (^*u*^mean). **a**, **b** boundary layer flow development along the S-plate in the absence of a ducted fan and (**c**), (**d**) boundary layer flow ingestion in the ducted fan under high- and low-thrust conditions, respectively. **a–d** Color bar represents the normalized mean velocity field (*u*_mean_/*U*_*∞*_), where higher values (in red) indicate regions of higher mean velocity, and lower values (in blue) represent regions of reduced mean velocity.

**Fig. 6:**
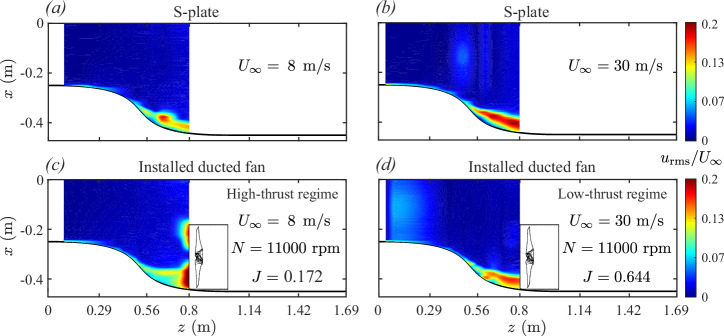
Evolution of the two-dimensional root-mean-square velocity field (^*u*^rms). **a**, **b** boundary layer flow development along the S-plate in the absence of a ducted fan and (**c**), (**d**) boundary layer flow ingestion in the ducted fan under high- and low-thrust conditions, respectively. **a**–**d** Color bar represents the normalized root-mean-square velocity field (*u*_rms_/*U*_*∞*_), where higher values (in red) indicate regions of higher rms velocity, and lower values (in blue) represent regions of reduced rms velocity.

Figures 5 and 6 show velocity contour maps illustrating the boundary layer flow ingestion process for the operational BLI ducted fan, comparing high-thrust and low-thrust conditions against the baseline S-plate configuration. These velocity maps display mean velocity *u*_mean_ (see Fig. 5) and root-mean-square velocity *u*_rms_ (see Fig. 6) distributions, highlighting the influence of fan thrust levels on the incoming boundary layer flow. The mean velocity field represents the steady-state characteristics of the flow, capturing the bulk momentum, while the root-mean-square velocity field illustrates the intensity of unsteady components that arise from the interaction between the boundary layer and the rotating fan blades.

In the baseline S-plate configuration, where the ducted fan is absent, the velocity fields reveal similar boundary layer flow development for both axial inflow velocities, i.e., *U*_*∞*_ = 8 and 30 m/s (see Fig. 5a, b for mean velocity and Fig. 6a, b for root-mean-square velocity). The mean velocity fields indicate that the boundary layer develops gradually along the concave curvature of the S-plate (0.5 ≤ *z* ≤ 0.8 m). This boundary layer growth is driven by curvature-induced flow distortion caused by the adverse pressure gradient effect, which promotes the formation of three-dimensional, stretched longitudinal vortices. These flow features, characteristic of turbulent boundary layers over concave surfaces, result from centrifugal instabilities analogous to Taylor–Görtler vortices^48^. The extent of boundary layer development in the S-plate setup also illustrates how a non-operational ducted fan, if installed, would be partially immersed in this boundary layer. The velocity distribution in the contour plots indicates that approximately one-quarter of the ducted fan would lie within the adverse pressure gradient flow, with the boundary layer measuring around 70 mm in thickness at the intended installation location, as reported by Ahmed et al.^11^. The root-mean-square (rms) velocity fields indicate low turbulence levels concentrated near the wall, further confirming the dominance of curvature-induced effects. The baseline configuration provides a reference for evaluating how the presence of an operational ducted fan modifies the curvature-induced distorted flow field under high- and low-thrust conditions.

When the installed (or BLI) ducted fan operates under high-thrust conditions, significant alterations are observed in both mean velocity *u*_mean_ (see Fig. 5c) and root-mean-square velocity *u*_rms_ (see Fig. 6c) fields. The velocity maps reveal two distinct flow features. The first is the development of a distorted boundary layer along the concave region of the S-plate (0.5 ≤ *z* ≤ 0.8), consistent with the baseline configuration (see Figs. 5a and 6a). This flow feature is driven by curvature-induced effects, where the adverse pressure gradient contributes to the boundary layer growth and localized turbulent fluctuations. The second feature is a prominent region observed in the immediate upstream of the operational ducted fan, characterized by elevated mean velocity (see Fig. 5c) and intensified turbulent unsteadiness (see Fig. 6c). This fan-induced upstream effect significantly modifies the characteristics of an incoming adverse pressure gradient boundary layer. The interaction of the two distortion mechanisms due to curvature-induced adverse pressure gradient and fan-induced upstream effect fundamentally alters the ingestion mechanisms. The elevated mean velocities in the immediate upstream region indicate intensified bulk momentum, while the elevated rms velocities reveal amplified unsteady flow fluctuations in the turbulent vortical structures. The rms velocity plot further highlights the pronounced influence of the fan-induced upstream effect on the ingestion process. The adverse pressure boundary layer is further distorted and deflected as it is drawn into the ducted fan, exposing the entire blade span to high-momentum, highly unsteady, turbulent flow.

In contrast, the low-thrust regime exhibits a different flow ingestion behavior, where the curvature-induced flow distortion is relatively less affected by the fan-induced upstream flow distortion. As shown in Figs. 5d and 6d, the velocity fields closely resemble those observed in the baseline S-plate configuration, indicating minimal fan-induced upstream effect. The interaction between the fan-induced upstream flow and the curvature-induced adverse pressure gradient boundary layer flow is significantly reduced, resulting in limited distortion and deflection of the incoming flow. The velocity fields suggest a weak interaction between the fan and the boundary layer, with only a small portion of the blade span exposed to the adverse pressure gradient boundary layer flow under low-thrust conditions. Consequently, the bulk momentum and turbulence levels near the fan remain comparable to those in the baseline S-plate configuration (without the ducted fan).

The comparison of velocity distributions in low- and high-thrust regimes reveals significant differences in the boundary layer flow ingestion mechanisms. Under high-thrust conditions, the strong coupling between fan-induced and curvature-induced effects intensifies ingestion, redistributing boundary layer momentum and turbulence. In contrast, low-thrust conditions exhibit weaker fan-induced effects, with flow ingestion dominated by curvature-induced flow distortion and minimal contribution from the fan-induced upstream flow distortion.

#### Pressure field

The following text discusses the pressure field along the curved S-plate for high-thrust (*J* = 0.172, *U*_*∞*_ = 8 m/s, *N* = 11,000 rpm) and low-thrust (*J* = 0.644, *U*_*∞*_ = 30 m/s, *N* = 11,000 rpm) BLI cases, compared against the baseline S-plate configuration (without the ducted fan) under similar axial inflow conditions (*U*_*∞*_ = 8 and 30 m/s).

**Fig. 7:**
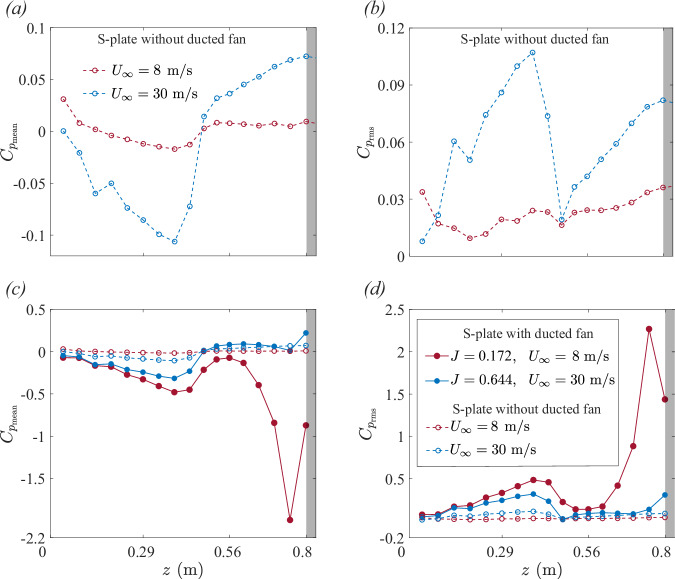
Static pressure distribution along the S-plate. **a** Mean (${C}_{{p}_{{\rm{mean}}}}$) and **b** root-mean-square (${C}_{{p}_{{\rm{rms}}}}$) pressure coefficient comparison between *U*_∞_ = 8 m/s and 30 m/s for the baseline S-plate without a ducted fan. **a** Mean (${C}_{{p}_{{\rm{mean}}}}$) and **b** root-mean-square (${C}_{{p}_{{\rm{rms}}}}$) pressure coefficient comparison between the installed ducted fan and the baseline S-plate configuration for low- and high-thrust conditions, with the fan operating at a rotational speed of *N* = 11,000 rpm. **a***–***d** Red dashed line represents the baseline S-plate configuration at *U*_*∞*_ = 8 m/s, blue dashed line represents the baseline S-plate configuration at *U*_*∞*_ = 30 m/s, solid red line represents the installed ducted fan at *J* = 0.172, and solid blue line represents the installed ducted fan at *J* = 0.644. The gray area represents the location of the ducted fan region.

Figure 7 presents the mean and root-mean-square pressure distributions along the S-plate for the operational BLI ducted fan under low- and high-thrust conditions, compared to the baseline S-plate configuration. In the baseline S-plate configuration (without the ducted fan), the mean pressure coefficient (${C}_{{p}_{{\rm{mean}}}}$) exhibits a smooth gradient along the S-plate surface, indicative of steady boundary layer growth for both the velocities *U*_*∞*_ = 8 and 30 m/s (see Fig. 7a). In the upstream convex region (0 ≤ *z* ≤ 0.3 m), a favorable pressure gradient accelerates the flow, reducing static pressure and resulting in a thin boundary layer. As the flow enters the concave region (0.3 ≤ *z* ≤ 0.8 m), an adverse pressure gradient causes a gradual increase in mean pressure, signifying boundary layer thickening as the flow decelerates. The root-mean-square pressure coefficient (${C}_{{p}_{{\rm{rms}}}}$) in this configuration remains low throughout the S-plate, reflecting minimal unsteady flow components (see Fig. 7b). This effect of curvature-induced adverse pressure gradient is more pronounced at *U*_*∞*_ = 30 m/s in comparison to the *U*_*∞*_ = 8 m/s. The absence of the ducted fan ensures that the flow dynamics are primarily influenced by curvature-induced steady, quasi-inviscid effects such as large-scale stretched vortices. These steady pressure variations align with velocity field observations in Figs. 5a, b and 6a, b, confirming stable and gradual curvature-induced boundary layer growth with flow distortion.

In the high-thrust regime, the installed ducted fan substantially modifies the pressure characteristics on the curved S-plate. The mean pressure coefficient (${C}_{{p}_{{\rm{mean}}}}$) exhibits a pronounced low-pressure zone in the immediate upstream of the ducted fan, at *z* ≈ 0.78 m (see Fig. 7c). This sharp drop in mean pressure reflects a strong fan-induced suction effect, which amplifies the ingestion of high-momentum boundary layer structures. The steep adverse pressure gradient associated with this fan-induced suction effect redistributes flow momentum, and draws high-momentum boundary layer structures toward the ducted fan, thereby significantly amplifying the ingestion process. The root-mean-square pressure coefficient (${C}_{{p}_{{\rm{rms}}}}$) distribution reveals elevated pressure fluctuations concentrated in the immediate upstream of the ducted fan (see Fig. 7d). These elevated pressure fluctuations indicate the ingestion of highly unsteady turbulent structures into the fan, imposing substantial unsteady aerodynamic loading on the blades. The dynamic interaction between the fan-induced suction effect and curvature-induced adverse pressure gradient significantly amplifies turbulence ingestion, redistributing boundary layer momentum and turbulence across a substantial portion of the blade span.

Under low-thrust conditions, the pressure characteristics along the curved S-plate for the installed ducted fan configuration exhibit considerably weaker fan-induced effects compared to the high-thrust conditions. The mean pressure coefficient ${C}_{{p}_{{\rm{mean}}}}$ shows only a mild pressure drop in the immediate upstream of the ducted fan (see Fig. 7c), indicating the absence of a strong fan-induced suction effect. Similarly, the root-mean-square pressure coefficient ${C}_{{p}_{{\rm{rms}}}}$ reveals significantly lower pressure fluctuations in the immediate upstream of the ducted fan, with fluctuations remaining concentrated near the wall (see Fig. 7d). These observations in the pressure characteristics suggest that the flow ingestion process in the low-thrust condition is primarily governed by the curvature-induced adverse pressure gradient effect, with minimal contribution from the fan-induced suction effect. The absence of a pronounced fan-induced suction effect limits the redistribution of boundary layer momentum and turbulence. Consequently, only a small portion of the blade span, primarily near the tip, interacts with the boundary layer flow, leading to reduced ingestion and minimal unsteady aerodynamic loading on the blades. The weak coupling between the fan-induced and curvature-induced effects ensures that the ingestion mechanism remains less intense compared to the high-thrust regime.

### Aeroacoustics assessment

The far-field noise radiation from the BLI ducted fan is considerably more complex than that of open (non-ducted) configurations. This increased complexity arises from the simultaneous contributions of two distinct noise sources, i.e., duct-induced noise and fan-induced noise. Additionally, the interaction between the incoming turbulent boundary layer flow and the ducted fan introduces yet another layer of complexity, further complicating the overall noise radiation characteristics of BLI ducted fan systems.

**Fig. 8:**
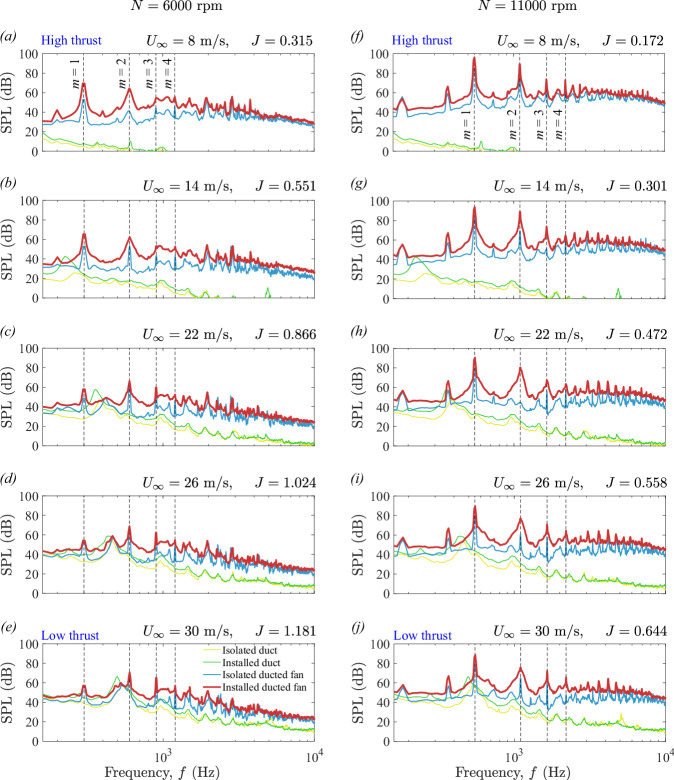
Sound pressure level (SPL) comparison of ducted fan configurations (see Table 1 and Fig. 4) at constant fan rotational speeds (*N*) of 6000 and 11,000 rpm, covering low- and high-thrust regimes. Measurements were captured at a microphone position of *θ* = 90°. Here, *m* = 1 corresponds to the blade passing frequency (BPF), and *m* = 2, 3, 4. . . the first, second, third, fourth harmonics, and so on. **a**–**j** Yellow line represents the isolated duct, the green line represents the installed duct, the blue line represents the isolated ducted fan, and the brick red line represents the installed ducted fan.

Figure 8 presents the individual noise contributions from various configurations, including the isolated duct, the installed duct, and the isolated ducted fan at fan rotational speeds (*N*) of 6000 and 11,000 rpm. These contributions are then compared with the noise spectra of the BLI ducted fan, i.e., installed ducted fan. The comparisons are highlighted across a range of fan thrust regimes, including low- and high-thrust levels, based on the measurements acquired at the microphone position of *θ* = 90°(see Fig. 3). The plots for various thrust regimes are shown for axial inflow velocities (*U*_*∞*_) of 8, 14, 22, 26, and 30 m/s, which correspond to advance ratios (*J*) of 0.315, 0.551, 0.866, 1.024, and 1.181 at *N* = 6000 rpm (see Fig. 8a–e), and 0.172, 0.301, 0.472, 0.558, and 0.644 at *N* = 11,000 rpm (see Fig. 8f, j). The nature of the complex interaction of the incoming turbulent boundary layer flow with the acoustic fields of the duct and rotating fan (i.e., duct and fan aeroacoustics) varies across fan thrust regimes, characterized by the haystacking phenomenon^11^. The term haystacking describes a phenomenon in which spectral humps and spectral broadening of tonal components appear within the noise spectrum^49^. Two types of haystacking phenomena were observed in the far-field noise data in Fig. 8, namely the fan and duct haystacking, both driven by the fan thrust levels. In the rest of this section, the mechanism behind each type of haystacking will be investigated.

When the fan is operating in the high-thrust regime, i.e., *U*_*∞*_ = 8 m/s and *J* = 0.315 at *N* = 6000 rpm (see Fig. 8a) and *U*_*∞*_ = 8 m/s and *J* = 0.172 at *N* = 11,000 rpm (see Fig. 8f), the duct’s contribution to the noise radiation is minimal. The noise spectrum of the installed ducted fan is thus primarily influenced by the interaction between the incoming turbulent boundary layer flow and the fan aeroacoustics. This interaction highlights a key feature of turbulence ingestion in open (non-ducted) fans, where the rotating blades chop off incoming turbulent flow structures, leading to a process known as the fan haystacking phenomenon^5^. This interaction mechanism under an adverse pressure gradient features a suction (or negative pressure) effect in the immediate upstream region of the ducted fan (see Fig. 7c, d), which amplifies the ingestion of high-momentum (see Fig. 5c), highly unsteady (see Fig. 6c) boundary layer vortical structures. As these turbulent structures are continuously drawn into the fan, they undergo intense chopping by the large portion of rotating blades’ sections (Fig. 6c), introducing additional unsteady aerodynamic loading and significantly amplifying turbulence levels in the flow. Consequently, this enhanced turbulence results in a fan haystacking phenomenon characterized by spectral humps and spectral broadening of tonal components. The spectral humps, observed between the second (*m* = 3) and third (*m* = 4) harmonics, as well as between the third (*m* = 4) and fourth (*m* = 5) (see Fig. 8a, f), are the typical features of open (or non-ducted) fans/propellers ingesting a turbulent boundary layer flow, a phenomenon well-documented in the literature, such as Murray et al.^5^. The spectral broadening, particularly noticeable at the blade passing frequency (BPF) (*m* = 1) and the first harmonics (*m* = 2) (see Fig. 8a, f), is the typical feature when the acoustic field interacts with a turbulent vortical field, as illustrated by Glegg and Devenport^13^ and Huang^49^.

Conversely, when the fan is operating in the low-thrust regime, i.e., *U*_*∞*_ = 30 m/s and *J* = 0.1. 181 at *N* = 6000 rpm (see Fig. 8e), and *U*_*∞*_ = 30 m/s and *J* = 0.644 at *N* = 11,000 rpm (see Fig. 8j), the haystacking mechanism differs from that observed in the high-thrust regime (see Fig. 8a, f). The mechanism of chopping of incoming vortices by a rotating fan also differs, as the incoming turbulent boundary layer flow is ingested into the ducted fan without undergoing a strong suction effect in the immediate upstream region of the ducted fan (see Fig. 7c, d). The absence of a pronounced fan-induced suction effect limits the redistribution of boundary layer momentum (see Fig. 5d) and turbulence (see Fig. 6d), confining the interaction primarily to the blade tip region. As these turbulent structures are continuously drawn into the fan, they undergo relatively weak chopping by the small portion of the rotating blades (see Fig. 6d), resulting in reduced ingestion and minimal unsteady aerodynamic loading relative to the high-thrust regime. However, the contribution of the duct to the noise spectrum of the installed ducted fan becomes more pronounced in the low-thrust regime, altering the characteristics of the haystacking phenomenon. This enhanced noise contribution from the duct is attributed to the duct’s blunt-trailing-edge-induced vortex shedding, which excites the duct’s planar acoustic field (see Fig. 2b), as reported by Ahmed et al.^11^. Although some of the planar wave energy stays contained within the duct, a portion escapes through the open ends, contributing to the far-field noise radiation^11,50^. The complex interaction between the incoming turbulent flow and the duct’s acoustic field results in duct haystacking. The noise spectrum for an installed duct is broader than that of the isolated duct at the first harmonic (*m* = 2) (see Fig. 8e, j). This spectral broadening in the installed duct configuration is attributed to duct haystacking—a different phenomenon from fan haystacking—resulting from the interaction between the duct’s acoustic field and the incoming turbulent vortical field. The enhanced contribution of duct in both the isolated and installed configuration significantly modifies the fan haystacking features that are prominent in the high-thrust regime. The noise features at low-thrust levels, characterized by spectral broadening and humps, are not solely due to the fan haystacking but arise from the combined effects of fan aeroacoustics, duct aeroacoustics, and the associated haystacking phenomenon. The alteration in the noise spectrum of the installed ducted fan is indicative of the complex interaction between the duct, the rotating fan, and the incoming turbulent boundary layer, leading to distinct noise characteristics in the low-thrust regime compared to the high-thrust regime.

The noise characteristics of a BLI ducted fan at *N* = 11,000 rpm (see Fig. 8f, j) differ significantly from those observed at *N* = 6000 rpm (see Fig. 8a–e). First, the increase in tip Mach number (*M*_tip_) at *N* = 11,000 rpm results in increased noise levels across the entire frequency range compared to the *N* = 6000 rpm. Additionally, at *N* = 11,000 rpm, the distribution of noise across frequencies changes noticeably. The low- and mid-frequency ranges, primarily dominated by the blade passing frequency (BPF) and its harmonics, become more pronounced compared to the high-frequency range, where fan self-noise contributes more prominently to the overall noise characteristics. This effect is particularly evident at high thrust levels (see Fig. 8f). Furthermore, the results indicate that at *N* = 11,000 rpm, fan-induced noise has a significantly stronger impact on the overall noise profile compared to duct-induced noise across the entire range of advanced ratios, particularly in the high-thrust regime. In contrast to the *N* = 6000 rpm case, the haystacking phenomenon—evident as spectral humps at the fundamental BPF (*m* = 1) and harmonics (*m* = 2 to 4)—is more pronounced at *N* = 11,000 rpm. This comparison of noise components demonstrates that the interaction between the incoming turbulent boundary layer flow and the ducted fan is the main source of the observed haystacking at two distinct fan rotational speeds.

**Fig. 9:**
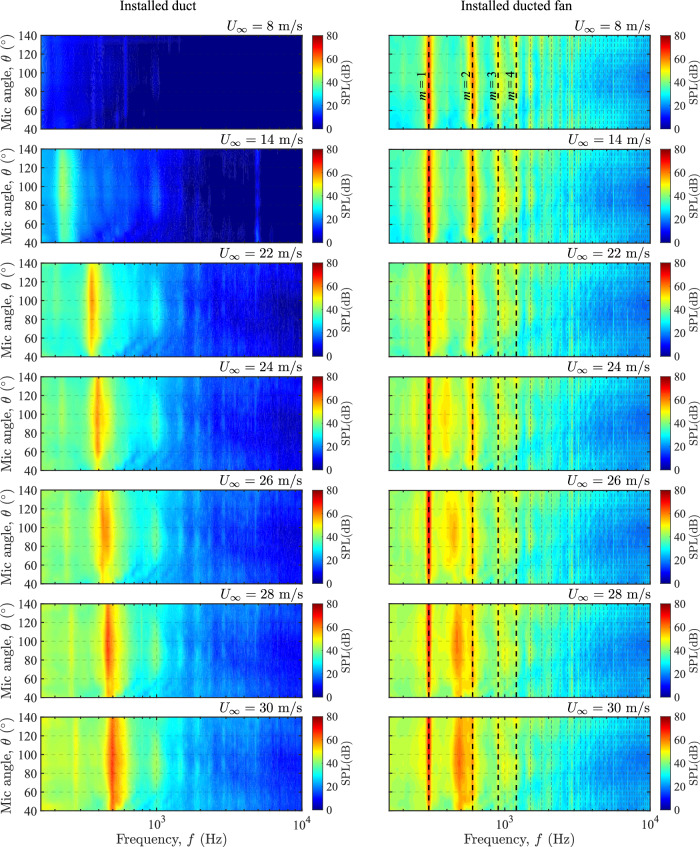
Comparison of noise contour maps in installed configurations for duct and ducted fans (see Table 1 and Fig. 4) at a fan rotational speed (*N*) of 6000 rpm for a wide range of fan thrust regimes. Here, *m* = 1 corresponds to the blade passing frequency (BPF), and *m* = 2, 3, 4. . . the first, second, third, fourth harmonics, and so on. colorbar represents the sound pressure level (SPL).

To further understand the influence of complex interaction between the incoming turbulent boundary layer flow, fan aeroacoustics, and duct aeroacoustics on the haystacking phenomenon, a noise contour map is plotted in Fig. 9. These results were obtained for the installed ducted fan across a wide range of thrust regimes, defined by advance ratios (*J*) via changing incoming axial flow velocities (*U*_*∞*_) of 8–30 m/s, and covering a wide range of microphone angles (*θ*) from 40° to 140°. These figures compare the noise characteristics of the installed ducted fan against the installed duct. While the fan aeroacoustics, i.e., noise associated with blade passing frequency (BPF) (*m* = 1) and the associated harmonics (*m* = 2, 3, 4, . . . ), is noticeable throughout the thrust regimes, duct aeroacoustics become more apparent and vary with the axial incoming velocity in the low-thrust regimes. The fan and duct haystacking phenomenon in the form of tonal spectral broadening is observed throughout the thrust regime. On the other hand, the generation of duct aeroacoustics and associated haystacking, alters the overall noise spectra in the low-thrust regimes.

This analysis shows how different thrust levels during forward flight can change the nature of the far-field noise radiation produced by the BLI ducted fan, highlighting the complex challenges in designing and optimizing embedded propulsion systems to achieve zero-emission sustainable aviation.

### Psychoacoustics assessment

The psychoacoustic assessment of a BLI ducted fan provides valuable insights into the perceived noise characteristics and their potential influence in terms of noise annoyance.

**Fig. 10:**
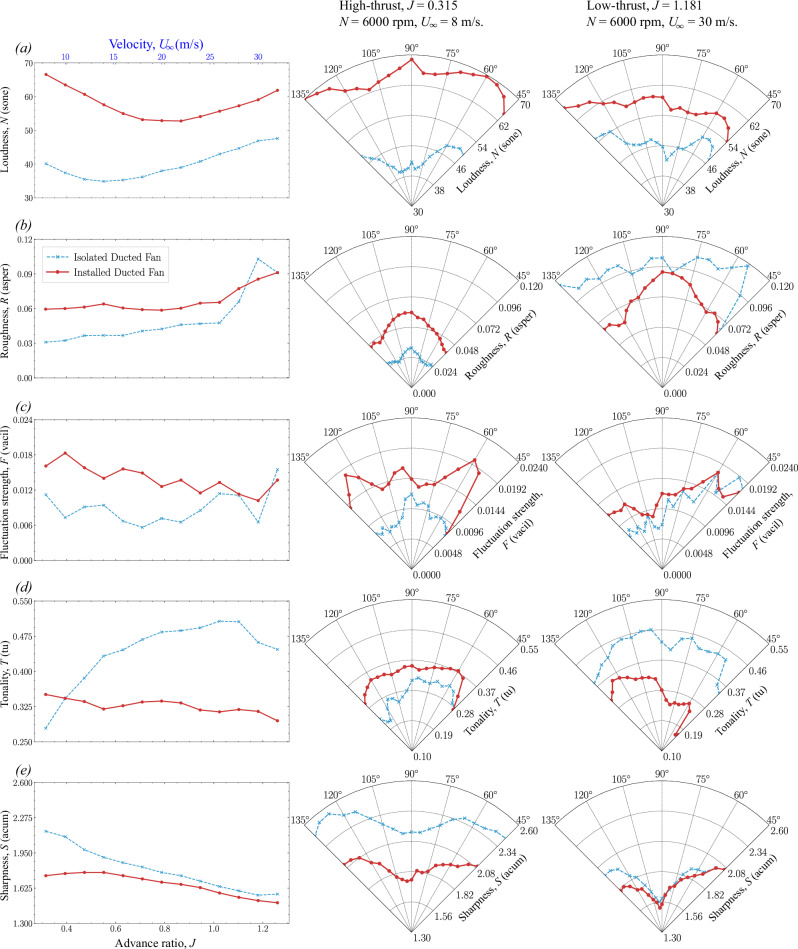
Sound quality metrics (SQMs) comparison of isolated and installed ducted fan configurations (see Table 1 and Fig. 4) at *N* = 6000 rpm. The left-hand side column shows the amplitude of SQMs over a range of advance ratios *J* at *θ* = 90°. The center and right-hand side columns show the directivity of SQMs (at 45° < *θ* < 135°) in the high-thrust and low-thrust regimes, respectively. **a**–**e** Dashed blue line with asterisks represents the isolated ducted fan, and the solid brick red line with circles represents the installed ducted fan.

**Fig. 11:**
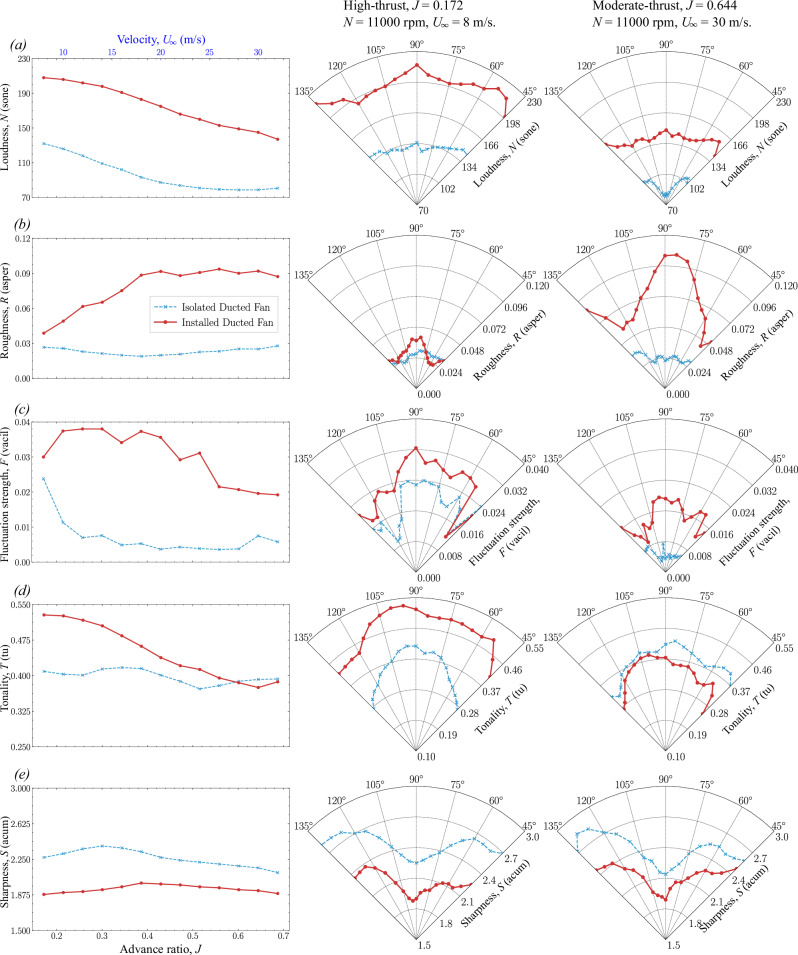
Sound quality metrics (SQMs) comparison of isolated and installed ducted fan configurations (see Table 1 and Fig. 4) at *N* = 11,000 rpm. The left-hand side column shows the amplitude of SQMs over a range of advance ratios *J* at *θ* = 90°. The center and right-hand side columns show the directivity of SQMs (at 45° < *θ* < 135°) in the high-thrust and low-thrust regimes, respectively. **a**–**e** Dashed blue line with asterisks represents the isolated ducted fan, and the solid brick red line with circles represents the installed ducted fan.

**Fig. 12:**
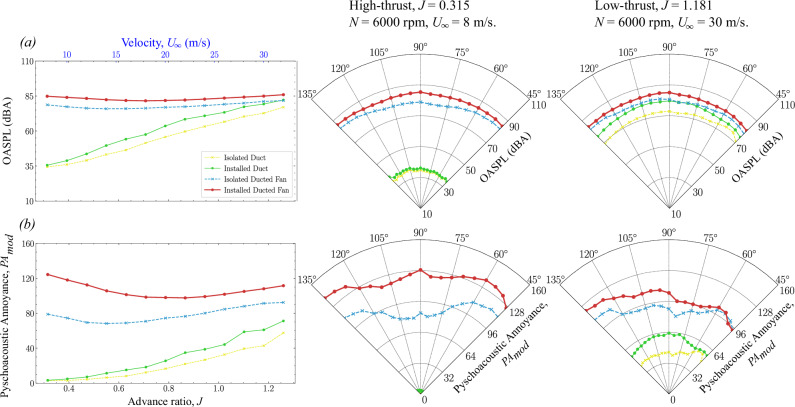
Overall A-weighted sound pressure level (O*A*SPL) and psychoacoustic annoyance (PA_mod_) comparison of isolated and installed ducted fan configurations (see Table 1 and Fig. 4) at *N* = 60,000 rpm. The left-hand side column shows the amplitude of O*A*SPL and PA_mod_ over a range of advance ratios *J* at *θ* = 90°. The center and right-hand side columns show the directivity of O*A*SPL and PA_mod_ (at 45° < *θ* < 135°) in the high-thrust and low-thrust regimes, respectively. **a** and (**b**) Yellow line represents the isolated duct, green line represents the installed duct, blue line represents the isolated ducted fan, and brick red line represents the installed ducted fan.

**Fig. 13:**
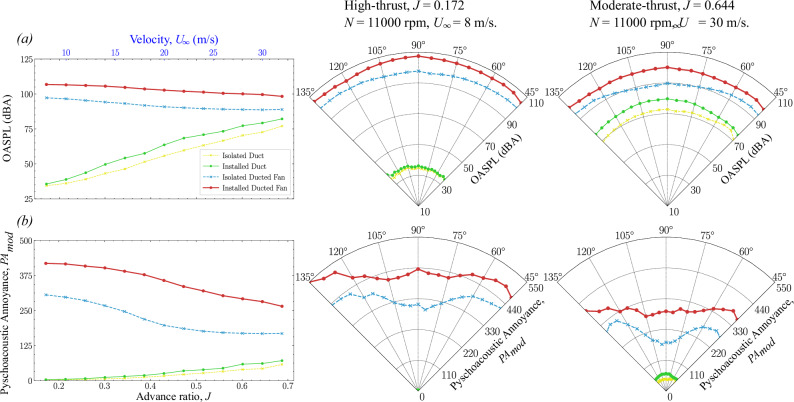
Overall A-weighted sound pressure level (O*ASPL*) and psychoacoustic annoyance (PA_mod_) comparison of isolated and installed ducted fan configurations (see Table 1 and Fig. 4) at *N* = 110,000 rpm. The left-hand side column shows the amplitude of O*A*SPL and PA_mod_ over a range of advance ratios *J* at *θ* = 90°. The center and right-hand side columns show the directivity of of O*A*SPL and PA_mod_ (at 45° < *θ* < 135°) in the high-thrust and low-thrust regimes, respectively. **a** and (**b**) Yellow line represents the isolated duct, green line represents the installed duct, blue line represents the isolated ducted fan, and brick red line represents the installed ducted fan.

Sound quality metrics (SQMs) for both isolated and installed configurations are presented at two fixed fan rotational speeds of *N* = 6000 rpm and *N* = 11,000 rpm in Figs. 10 and 11, respectively. These SQMs include Loudness (*N*), Roughness (*R*), Fluctuation strength (*F*), Tonality (*T*), and Sharpness (*S*), which are critical for understanding how noise is perceived by humans. These SQMs are analyzed across a wide range of advance ratios (*J*) and thrust regimes, including low- and high-thrust levels, based on measurements acquired from microphone positions (*θ*) between 45° and 135° (see Fig. 3b). The corresponding overall *A*-weighted sound pressure level (O*A*SPL) and psychoacoustic annoyance (PA_mod_) are also calculated to provide an overall sense of perceived noise annoyance (see Figs. 12 and 13).

The directivity plots for the SQMs, O*A*SPL, and PA_mod_ are then compared among various fan thrust regimes. For *N* = 6000 rpm, the comparisons are made between low-thrust (*U*_*∞*_ = 30 m/s, *J* = 1.1815) and high-thrust (*U*_*∞*_ = 8 m/s, *J* = 0.315) conditions. The SQM results are shown in Fig. 10, while the O*A*SPL and PA_mod_ results are presented in Fig. 12. For *N* = 11,000 rpm, the comparisons are made between low-thrust (*U*_*∞*_ = 30 m/s, *J* = 0.644) and high-thrust (*U*_*∞*_ = 8 m/s, *J* = 0.172) conditions. The SQM results are shown in Fig. 11, and the O*A*SPL and PA_mod_ results are shown in Fig. 13.

As discussed in the aerodynamics and aeroacoustic assessments, the mechanisms of far-field noise radiation from the BLI ducted fan are driven by the fan thrust regimes, which influence the interaction among the incoming turbulent boundary layer flow and the fan and duct aeroacoustics. The perception of noise resulting from these interactions is governed by the haystacking phenomenon, which contributes uniquely to the overall annoyance experienced by listeners. The detailed mechanisms behind each of the SQMs, as well as the O*A*SPL and PA_mod_, are analyzed and explained in the following text.

#### Loudness

Loudness (*N*) quantifies the perceived intensity of sound, which is directly related to human annoyance and discomfort. The perception of Loudness in the BLI ducted fan is influenced by several factors: (i) the tonal components, such as Blade Passing Frequencies (BPF) and associated harmonics, and the broadband noise components generated by fan aeroacoustics, (ii) the planar tonal and broadband noise components originating from duct aeroacoustics, and (iii) the complex interaction between the incoming turbulent boundary layer and the fan and duct aeroacoustics.

In the isolated ducted fan configuration, the interaction between the incoming turbulent boundary layer flow and the aeroacoustics of the fan and duct is absent across all thrust regimes. As a result, the Loudness perception of noise in this configuration is solely influenced by the interaction of sound intensity generated by the fan and duct. When the fan is operating in the high-thrust regime (see Figs. 10a and 11a), Loudness is primarily driven by the fan aeroacoustics, as the duct’s contribution (i.e., duct aeroacoustics) is minimal (see Fig. 8a, f). In these regimes, the tonal noise components, such as blade passing frequency (BPF) and the associated harmonics, dominate the perceived Loudness, with the duct exhibiting a negligible contribution. Conversely, when the fan is operating in the low-thrust (see Fig. 10a) and low-thrust (see Fig. 11a) regimes, the perception of Loudness changes significantly. In these regimes, Loudness is influenced by both fan and duct aeroacoustics, with the fan’s contribution being minimal and duct’s contribution becoming more pronounced (see Fig. 8e, j). The enhanced noise contribution from the duct is due to blunt-trailing-edge-induced vortex shedding-induced duct acoustics (see Figs. 2b, 8, and 9)^11^.

The installed ducted fan, on the other hand, exhibits relatively higher Loudness values as compared to the isolated ducted fan configuration throughout the thrust regime. The difference in Loudness between the isolated and installed ducted fan configurations is primarily driven by the (fan and duct) haystacking phenomenon, which occurs when the fan and duct aeroacoustics interact with the incoming turbulent boundary layer flow. When the fan is operating in the high-thrust regime (see Figs. 10a and 11a), the major contributor to Loudness is the fan haystacking phenomenon, with minimal contributions from duct haystacking (see Fig. 8a, f). The enhanced Loudness in the high-thrust regime is driven by the fan haystacking, where the enhanced chopping of incoming vortices (see Figs. 5c and 6c), due to suction (or high negative pressure) in the immediate upstream of the ducted fan see Fig. 7c, d), occurs as the vortices are ingested into the ducted fan by the rotating blades. As the duct’s contribution is minimal, it is anticipated that the Loudness in the high-thrust regime for the installed ducted fan is similar to that of open (or non-ducted) fans. Conversely, when the fan is operating in the low-thrust (see Figs. 10a and 11a) regimes, the major contributor to Loudness is the duct haystacking phenomenon, with minimal contributions from fan haystacking (see Fig. 8e, j). In these regimes, the interaction between the incoming turbulent boundary layer flow and the duct’s blunt-trailing-edge-induced vortex shedding becomes more pronounced, enhancing the noise contribution from the duct (see Figs. 2b, 8, and 9) and, consequently, leading to higher values of Loudness.

The directivity plots reveal that in the high-thrust regime (see Fig. 10a and 11a), the upstream region (*θ* < 90°) experiences higher Loudness, with a peak around 90°. This is attributed to the noise radiation in the plane of the duct lip. In the low-thrust regime (see Figs. 10a and 11a), Loudness decreases across all microphone positions, with higher values directed towards the downstream region (*θ* > 90°), due to the shift in noise contribution from the duct.

#### Roughness and fluctuation strength

Roughness (*R*) measures the perception of rapid sound modulation that occurs within the frequency range between 15 and 300 Hz, with a peak around 70 Hz. In contrast, the Fluctuation strength (*F*) measures the perception of slower modulations occurring at very low frequencies, up to about 20 Hz, with a peak around 4 Hz. Fluctuation strength is associated with turbulence and is perceived as a temporal factor^51^. The modulation phenomena due to the interaction among the fan aeroacoustics, duct aeroacoustics, and the incoming turbulent boundary layer flow seem to be captured on the frequency modulation ranges for Roughness and Fluctuation strength. These two metrics provide valuable insight into how temporal changes in sound, particularly in terms of modulation, influence human perception and discomfort associated with the noise generated by the BLI ducted fan system.

In the isolated ducted fan configuration, Roughness and Fluctuation strength are solely influenced by the modulation due to the interaction between the fan and duct aeroacoustics, as the incoming turbulent boundary layer is absent. It is important to note that as the fan is installed within the ducted fan with a certain distance from the duct inlet (see Fig. 2b), it is anticipated that the duct’s inlet distorted flow interacts with the fan resulting in a certain amount of Roughness and Fluctuation strength.

In comparison to the isolated ducted fan, the installed ducted fan configuration exhibits relatively higher Roughness and Fluctuation strength across the thrust regime. These metrics are primarily driven by the modulation due to the interaction among the fan aeroacoustics, duct aeroacoustics, and the incoming turbulent boundary layer flow. When the fan is operating in the high-thrust regime (see Figs. 10b, c and 11b, c), the installed ducted fan exhibits higher Roughness and Fluctuation strength compared to the isolated configuration. This difference diminishes as the fan moves towards the low-thrust (see Figs. 11b, c and 10b, c) regimes.

In the high-thrust regime (see Figs. 10b, c and 11b, c), the duct’s contribution is minimal, and hence the modulation is driven primarily by fan aeroacoustics, where the broadband noise from the incoming turbulent boundary layer flow is modulated with the blade passing frequency (BPF) and the associated harmonics, producing noticeable sidebands (see Fig. 8f). As the fan operates in low-thrust (see Figs. 11b, c and 10b, c) regimes, the duct’s contribution becomes more significant, and the interaction between the turbulent boundary layer flow and duct aeroacoustics alter the noise characteristics. In these regimes, sideband noise diminishes due to the duct haystacking, and the broadband noise modulated by the BPF becomes a dominant source of modulation, influencing both Roughness and Fluctuation strength.

The directivity plots (see Figs. 10b, c and 11b, c) reveal that in the installed ducted fan configuration, Roughness is concentrated in the fan’s rotational plane (*θ* = 90°), where the ducted fan interacts with the incoming turbulent boundary layer flow throughout the thrust regime. In contrast, Roughness tends to be more omnidirectional in the isolated configuration. For Fluctuation strength, the installed ducted fan exhibits additional symmetric lobes at ±30° from the fan’s rotational plane (*θ* = 90°), particularly in the high-thrust regimes. However, in the low-thrust regime, Fluctuation strength shifts upstream (*θ* < 90°), reflecting the changing noise modulation dynamics as duct aeroacoustics begin to dominate.

#### Tonality

The perception of narrowband tones within the noise spectrum is evaluated by Tonality (*T*). The interaction of the ducted fan with the incoming turbulent boundary layer flow can significantly alter the Tonality in the BLI ducted fan. The spectral broadening associated with the duct and fan haystacking (as observed in Fig. 8), causes alteration in the narrowband tones, and hence the Tonality.

In the isolated ducted fan configuration, where the interaction of a ducted fan with the incoming turbulent boundary layer flow is absent, the tonal components remain narrowband throughout the thrust regime. As a result, Tonality values are consistently higher in this configuration compared to the installed ducted fan, except at very high-thrust levels. At low-thrust (see Figs. 10d and 11d) regimes, Tonality is influenced by the presence of the duct’s blunt-trailing-edge vortex shedding, which induces significant low-frequency tonal noise at higher axial inflow velocities (see Figs. 2b, 8, and 9). This results in a noticeable increase in Tonality in the isolated ducted fan configuration.

Conversely, in the installed ducted fan setup, Tonality is relatively higher in the high-thrust regime (see Figs. 10d and 11d) but tends to decrease at low-thrust (see Figs. 10d and 11d) regimes. The reduction in Tonality in low-thrust regime is due to the complex interaction between fan and duct aeroacoustics, including the effects of duct haystacking. This interaction causes spectral broadening at the blade passing frequency (BPF) and the associated harmonics, which effectively reduces the calculated Tonality in the installed configuration at lower thrust levels (see Fig. 9).

The directivity plots reveal that in the installed ducted fan configuration, the incoming turbulent boundary layer flow attenuates the amplitude of tonal components across all microphone positions. Tonality tends to be directed toward the downstream region (*θ* > 90°) in low-thrust regime (see Figs. 11d and 10d) level, whereas, in the high-thrust regime, the isolated ducted fan configuration shows Tonality directed toward the fan’s rotational plane. In very high-thrust conditions, Tonality points upstream, highlighting how the interaction of fan and duct aeroacoustics with the incoming turbulent boundary layer flow can significantly alter the Tonality in the BLI ducted fan system.

#### Sharpness

Sharpness (*S*) refers to the contribution of the higher frequency components relative to the low frequencies to the spectral envelope of sound. The perception of Sharpness is closely linked to how the human ear interprets these frequency distributions, impacting overall sound quality and annoyance. Generally, sounds with a higher proportion of high-frequency content are perceived as sharper and often less pleasant to listen to^52^. As a rule of thumb, the low-frequency range can be assumed to extend up to 2 kHz, with frequencies above this threshold considered high. This point marks the transition where broadband noise begins to dominate over tonal components.

The isolated ducted fan configuration, where the incoming turbulent boundary layer flow is absent, shows minimal interaction between the flow and ducted fan. As a result, the tonal components in the low-frequency range are narrower, and broadband noise levels are lower. This lack of spectral broadening in the low-frequency range leads to relatively higher Sharpness values in the isolated ducted fan configuration. While the installed ducted fan reveals higher sound energy across the entire frequency range throughout the thrust regime, the difference in Sharpness between the two configurations becomes more pronounced in the high-thrust regime.

The spectral broadening effect observed in the installed configuration in the high-thrust regime (see Figs. 10e and 11e) is driven by the interaction between the ducted fan and the incoming turbulent boundary layer flow. This interaction mechanism produces a suction effect in the immediate upstream region of the ducted fan in the high-thrust regime. This suction effect, as discussed earlier (see Fig. 7c, d), enhances the chopping of incoming vortices (see Figs. 5c and 6c), when ingesting into the rotating fan, generating haystacking and broadband noise that extends into the lower frequency range. These additional broadband noise components contribute to the overall sound energy but decrease the perceived Sharpness by reducing the dominance of high-frequency components.

In the low-thrust (see Figs. 10e and 11e) regimes, the Sharpness values of both configurations tend to converge. This is due to the reduced interaction between the turbulent boundary layer and fan aeroacoustics, resulting in a more balanced sound energy distribution across the frequency range. The noise characteristics in this regime, characterized by spectral broadening and humps due to duct haystacking, are more evenly distributed between high and low frequencies, leading to similar Sharpness values for both configurations.

The directivity plots indicate that Sharpness is reduced across all microphone positions (45° < *θ* < 135°) due to the influence of the incoming turbulent boundary layer flow. Additionally, the contribution of high frequencies appears to be minimized in the fan’s rotational plane (*θ* = 90°) for both the installed and isolated ducted fan configurations, further influencing the calculated Sharpness in those regions.

#### OASPL and Psychoacoustic Annoyance

The distributions of *OASPL* and *PA*_*mod*_ for both configurations of ducted fan are shown in Figs. 12 and Fig. 13 for *N* = 6000 rpm and *N* = 11,000 rpm, respectively. As expected, the amplitude of O*A*SPL and PA_mod_ correlates directly with thrust, reaching its highest levels in the high-thrust regime and gradually decreasing in the low-thrust regimes. Interestingly, both O*A*SPL and PA_mod_ exhibit a similar trend, with their values reaching a minimum when the advance ratio *J* approaches the pitch-to-diameter ratio of 0.85. This effect is depicted in Fig. 12a. It is worth noting that the directivity trends of O*A*SPL and PA_mod_ closely align with those of Loudness. The quasi-similar patterns between O*A*SPL and PA_mod_ are likely due to the dominant influence of Loudness on the overall calculated perception of annoyance.

In the high-thrust regime (see the directivity plots in Figs. 12 and 13), the installed ducted fan configuration exhibits higher O*A*SPL and PA_mod_ values compared to the isolated configuration. This increase is primarily driven by the enhanced interaction between the incoming turbulent boundary layer flow and the fan aeroacoustics, characterized by the fan haystacking phenomenon, with minimal contributions from duct aeroacoustics.

In the low-thrust regimes (see the directivity plots in Figs. 12 and 13), the difference in O*A*SPL and PA_mod_ between the isolated and installed ducted fan configurations becomes more pronounced. The installed configuration displays a more complex noise signature due to the duct haystacking phenomenon, where the interaction between the duct aeroacoustics and the incoming turbulent boundary layer flow plays a more significant role.

### Key findings

This study provides a comprehensive assessment of the aerodynamic, aeroacoustic, and psychoacoustic characteristics of the boundary layer ingesting (BLI) ducted fan. By dissecting the complexities of physical interaction mechanisms in the BLI ducted fan, this framework enables the different contributions to noise and annoyance to be distinguished. The analyses cover a wide range of thrust regimes, highlighting the complex interactions that influence fan performance, noise radiation, and perceived annoyance.

The aerodynamic assessment identifies two distinct flow distortion mechanisms that govern the ingestion process: curvature-induced flow distortion, caused by the adverse pressure gradient along the concave curvature of the S-plate, and fan-induced flow distortion, driven by the upstream suction effect of the operational ducted fan. These two mechanisms interact and fundamentally alter the boundary layer ingestion process depending on the operating thrust regime.

In the high-thrust regime, the fan-induced suction effect dominates, creating a steep pressure gradient in the immediate upstream region of the ducted fan. This suction effect significantly redistributes the curvature-induced adverse pressure gradient distorted flow field, amplifying the ingestion process. The intensified interaction between the curvature- and fan-induced distorted flow fields exposes a large portion of the blade span to high-momentum turbulent structures. These flow interactions lead to substantial changes in boundary layer structures, affecting the fan aerodynamic loading characteristics.

In contrast, the low-thrust regime is characterized by a weaker fan-induced suction effect. The curvature-induced adverse pressure gradient boundary layer remains relatively undisturbed, exhibiting limited momentum redistribution and minimal turbulence amplification. The reduced interaction between the curvature- and fan-induced distortion fields results in a smaller portion of the blade span being exposed to relatively weak unsteady fan aerodynamic loading compared to the high-thrust condition.

The aeroacoustic assessment revealed distinct noise characteristics across thrust regimes, directly linked to the aerodynamic ingestion mechanisms. In the high-thrust regime, the fan aeroacoustics and the associated fan haystacking phenomenon dominate the noise spectrum, characterized by spectral humps and broadening of blade passing frequency (BPF) harmonics. This results from the intensified ingestion process driven by the fan-induced suction effect, which amplifies the ingestion of high-momentum turbulent structures across a large portion of the blade span. The enhanced chopping of these turbulent vortices by the rotating blades leads to increased unsteady aerodynamic loading, directly contributing to elevated sound pressure levels (SPLs) and stronger tonal and broadband noise radiation.

Conversely, in the low-thrust regime, where the ingestion process is primarily governed by curvature-induced flow distortion, the duct haystacking phenomenon becomes more pronounced. The absence of a strong fan-induced suction effect limits the redistribution of boundary layer momentum, resulting in weaker ingestion of high-energy turbulent structures. As a result, duct aeroacoustics and the associated duct haystacking, driven by the interaction between the incoming turbulent boundary layer flow and the duct’s blunt-trailing-edge-induced vortex shedding-plays a more significant role in noise generation. The interaction between these curvature-induced flow distortions and the duct acoustic field alters the noise signature, leading to distinct spectral characteristics compared to the high-thrust regime.

The integration of psychoacoustics into the aeroacoustics design of boundary layer ingestion (BLI) ducted fans is crucial because it provides valuable insights into its perceived noise characteristics, and what are the most efficient design changes allowing significant changes in noise annoyance. As shown in the SQM’s results, variation in sound quality features is linked to the primary noise generation mechanisms across the thrush regime. For example, in an isolated ducted fan, fan-aeroacoustics dominate in the high-thrust regime, whereas both fan and duct acoustics are significant noise contributors in the low-thrust regime. When a BLI surface is attached to the ducted fan, the main psychoacoustic effect is an increase in loudness due to duct and fan haystacking phenomena throughout the thrust regime. BLI phenomena, such as the spectral broadening effect of blade passing frequencies and haystacking effects over narrow frequency areas, lead to changes in the timbre (spectral composition) of the emitted sound. These changes can potentially increase annoyance levels among listeners and can be evaluated using various sound quality metrics (SQMs) and perceived annoyance models.

Incorporating psychoacoustic considerations into the design process ensures that the resulting propulsion systems not only perform well aerodynamically but also minimize environmental noise impact. The psychoacoustic assessment demonstrates that the incoming turbulent boundary layer flow significantly influences the sound quality and perceived annoyance of a BLI ducted fan noise across various thrust regimes. This holistic approach to design can lead to greater acceptance and satisfaction among users, ultimately contributing to the success of new aerial transportation technologies.

Overall, this study highlights the importance of understanding the interactions between aerodynamic, aeroacoustic, and psychoacoustic factors in the BLI ducted fan systems. The findings indicate that both duct and fan aeroacoustics must be considered across a wide range of fan operational thrust regimes during the design and operation of BLI ducted fans. This integrated approach aims to provide valuable insights for developing advanced noise reduction strategies and enhancing the acoustic comfort of next-generation aircraft.

## Methods

The methods employed for the assessment of aerodynamics, aeroacoustics, and psychoacoustics associated with the BLI ducted fan are briefly summarized below. Detailed descriptions are provided in the “Results” section under subsections “Experimental set up”, “Analysis framework”, and “Diagnostic tools”.

### Wind tunnel and BLI ducted fan test set-up

The experimental campaign was carried out in the University of Bristol’s aeroacoustics wind tunnel. A custom test rig was used, featuring an electric ducted fan mounted adjacent to a curved S-plate wall designed to simulate an adverse pressure gradient (see Fig. 3a, b). The configuration mimics a portion of an aircraft fuselage with a partially embedded fan, enabling the study of both aerodynamic performance and noise generation under realistic conditions. High-resolution flow (velocity and pressure field) and noise data were acquired using hot-wires, pressure taps, and far-field microphones, respectively (see Fig. 3b–d). Further details regarding the BLI setup, instrument placement, and data acquisition are discussed in the subsection “Experimental set-up” in the “Results and discussion” section.

### Analysis key parameters

An integrated analysis framework based on the source decomposition approach (see Fig. 4 and Table 1) was adopted to correlate aerodynamic behavior with aeroacoustic and psychoacoustic responses. Aerodynamic parameters (e.g., mean and root-mean-square velocity fields and pressure coefficients) were used to characterize the boundary layer development and the curvature- and fan-induced flow distortions. In parallel, the aeroacoustic analysis involved calculating sound pressure levels (SPL) and decomposing noise contributions into fan and duct components. Additionally, a suite of sound quality metrics (SQMs) such as Loudness, Roughness, Fluctuation Strength, Tonality, and Sharpness was employed to assess the perceptual impact of the noise. More comprehensive information on the analysis framework and key parameters is provided in the “Analysis framework” and “Diagnostic tools” subsections of the “Results and discussion” section.

## Data Availability

Data available on request from the authors.
